# Isoenergetic Replacement of Fat by Starch in Diets for African Catfish (*Clarias gariepinus*): Effect on Water Fluxes in the Gastro Intestinal Tract

**DOI:** 10.1371/journal.pone.0055245

**Published:** 2013-01-25

**Authors:** Till S. Harter, Johan A. J. Verreth, Leon T. N. Heinsbroek, Johan W. Schrama

**Affiliations:** Aquaculture and Fisheries Group, Wageningen University, Wageningen, The Netherlands; National Institute of Agronomic Research, France

## Abstract

The effect of an isoenergetic replacement of dietary fat by starch, on chyme characteristics and water fluxes in the gastro intestinal tract (GIT) was assessed. Adult African catfish (*Clarias gariepinus*) were fed a starch (SD) or fat (FD) diet and groups of fish were dissected at 2, 5 and 8 h after the consumption of a single meal. Chyme was collected quantitatively and was analysed for osmolality and dry matter (DM) content. Postprandial water fluxes were calculated, while using yttrium oxide (Y_2_O_3_) as an inert marker to account for the absorption of DM along the GIT. The largest differences in chyme characteristics between diets were observed in the stomach and decreased towards subsequent compartments. A high initial osmotic pressure was measured in the stomach for both diets (up to 498±2 mOsm kg^−1^) and was likely the driver for the endogeneous water influx to this compartment. Large additions of water were recorded to the stomach and proximal intestine for both diets and absorption of water took place in the mid- and distal intestine. Interestingly, the dietary treatment had an impact on water balance in the stomach and proximal intestine of the fish, but not in the mid- and distal intestine. A strong complementary relationship suggested that 59% of the water fluxes in the proximal intestine could be explained by previous additions to the stomach. Therefore, a higher dietary inclusion of starch led to a shift in water additions from the proximal intestine to the stomach. However, the sum of water additions to the GIT was not different between diets and was on average 6.52±0.85 ml water g^−1^ DM. The interactions between osmoregulation and digestion, in the GIT of fed freshwater fish, deserve further attention in future research.

## Introduction

Many physiological functions in fish, like respiration and absorption of nutrients demand large surfaces of permeable epithelia to enable active and diffusive transport processes. But this also increases the exposure of the animal to environmental conditions, such as salinity and pH, which may differ from the physiologically desired values. Electrochemical and osmotic gradients across the semipermeable membranes lead to unidirectional passive diffusion processes, which need to be counteracted actively to maintain homeostasis. Osmoregulation is the sum of regulatory processes that maintain the osmotic concentration of body fluids within the range of the physiological tolerance of the species [1]. Unlike their marine counterparts, freshwater (FW) fish are hyperosmotic compared to their environment. This means that they constantly lose ions to the environment and take up water by diffusion and intercellular leakage [1]. Processes of water balance take place in the skin, the gills, the kidneys and contrary to former beliefs [2], also in the gastro intestinal tract (GIT).

In FW species the role of the GIT in osmoregulation only becomes apparent during feeding, in relation to the uptake of dietary ions [3] and postprandial drinking [4], [5], [6], [7]. Due to its attractive simplicity, most studies on osmoregulation have been performed on starved animals and in FW fish this has led to the underestimation of the role of the GIT in osmoregulation. Under these circumstances it was acknowledged, that FW fish would cover their ion demand solely by branchial and renal uptake. Today it is recognized that this is only one part of the picture and that the uptake of dietary ions is an important pillar of the hyperosmotic strategy of FW fish [3].

In modern aquaculture, fish are fed extremely concentrated dry pelleted diets. It is assumed that from an evolutionary point of view, fish are not adapted to process such high dietary dry matter (DM) contents and that this places a physiological strain on the GIT [8]. Previous studies have reported a longer retention time of chyme in the stomach upon consumption of dry diets and that a high DM content of chyme would be counteracted by an increased addition of water to the GIT [7], [9]. These fluxes have been reported to be of both, endogeneous and exogeneous origin [6].

Chyme characteristics have been suggested to influence the water balance in the GIT. Bucking and Wood [10] investigated the water fluxes in the digestive tract of FW rainbow trout (*Oncorhynchus mykiss*) during the processing of a single meal, and detected large additions of water to the stomach and proximal intestine. The osmotic pressure generated in the stomach, upon consumption of the dry diet, has been proposed to drive the addition of endogeneous water from the interstitial fluid to the lumen. This assumption requires further substantiation. Furthermore, the effect of dietary characteristics on the kinetics of DM and ion concentration of the chyme, along the GIT has been described in previous research [11]. Therefore, in the present study, the water balance in the GIT was assessed in response to different dietary characteristics, which were expected to yield a difference in chyme osmolality and DM content during digestion. It was expected, that in line with previous findings, these differences in chyme characteristics would be reflected in altered water balance processes.

In the quest for sustainability, increasing amounts of plant ingredients are being included in aquaculture diets, as energy and protein carriers. For many farmed fish species, starch is being employed as a cheap source of non-protein energy. Its digestion in the GIT yields large amounts of osmotically active mono- and disaccharides. If the osmotic gradient across the epithelium drives the addition of water to the GIT, as proposed by Bucking and Wood [10], changes in the osmolality of chyme should be reflected in altered water fluxes. Therefore, the replacement of fat by starch is expected to increase the water additions to the GIT. Based on the methodology employed by Bucking and Wood [10], the present study assessed the postprandial water fluxes in the GIT by dissection of fish and chyme collection, using yttrium oxide (Y_2_O_3_) as an inert marker to account for the absorption of DM. Contrasts in dietary characteristics were created by the isoenergetic replacement of dietary fat by starch.

Large additions of endogeneous water to the GIT may lead to the rectal excretion of water with the faeces [10]. This is considered advantageous for hyperosmoregulators, but the implication of an increased osmotic challenge to the animals is yet unknown. The present study assessed, whether the replacement of dietary fat by starch led to an altered rectal excretion of water.

In light of the present gaps in knowledge, two diets with different sources of non-protein energy, i.e. by replacing fat versus starch in an isoenergetic way, were used to induce contrasts in dietary characteristics. The aim of the present research was to detect a causal relationship, between dietary characteristics, the resulting development of chyme characteristics in the GIT and processes of water balance.

## Materials and Methods

### 1.1 Ethics

This study was carried out in strict compliance with the Dutch legislation on animal experiments and the protocol was approved by the ethics committee for animal experiments of Wageningen University (DEC: 2009121.b).

### 1.2 Animals and housing

Adult African catfish (*Clarias gariepinus*) (*n* = 180), of mixed sex and average individual body weight of 941±195 g (Mean±SD.) were obtained from the experimental hatchery of Wageningen University, “De Haar Vissen” (Wageningen, The Netherlands). Fish were randomly stocked into twelve 120-litre aquaria, all connected to a single recirculation system (comprising a common water reservoir, a plate separator for solids removal and a trickling filter for gas exchange and NH_4_^+^ removal). Initial stocking density was approximately 118 kg m^−3^. The flow rate in the tanks was set to 7–8 L min^−1^; temperature was 27–28°C and a photoperiod of 12:12 h was maintained, with daybreak at 7.00 am. Water quality was monitored daily and measured values did not exceed the predefined limits: NH_4_-N, 7 mg L^−1^; NO_2_-N, 4 mg L^−1^; NO_3_-N 250 mg L^−1^; conductivity 3000–3500 μS cm^−1^ and pH 7–7.5. Whenever necessary, low pH was corrected by adding NaHCO_3_.

### 1.3 Diet preparation

Two extruded pelleted dry feeds were produced by Research Diet Services B.V. (Wijk bij Duurstede, The Netherlands). The experimental diets differed only in the source of non-protein energy. In the starch diet (SD) 300 g of gelatinized maize starch was added to 700 g of basal ingredient mixture, while in the fat diet (FD) this starch was replaced by an isoenergetic amount of vegetable oil (125 g) (Table 1). Therefore, the ratio of nutrients (including protein) to gross energy (GE) was the same for both diets. Due to the higher energy density of fat, FD was more concentrated compared to SD on a DM basis. It was chosen, not to overcome this by adding a filler such as cellulose, since an effect on water fluxes is difficult to exclude. After extrusion, the starch in SD expanded considerably, while no increase in volume was observed in FD. Therefore, the presented formulation resulted in floating pellets for SD and sinking pellets for FD. Yttrium oxide (Y_2_O_3_) was included in both diets to account for the absorption of DM along the GIT in subsequent calculations. Feeds were kept in cold storage (4°C) and were sieved (>1.5 mm), to remove dust and smaller particles prior to use. A representative sample of both diets was taken and stored at 4°C for analysis.

**Table 1:** Formulation and measured nutrient composition of experimental starch (SD) and fat diet (FD).

	Diet	
Diet formulation	SD^1^	FD^2^
	[g kg^−1^]	
Gelatinized maize starch	300.0	–
Palm oil	–	75.8
Soy oil	–	75.8
Fish meal (CP>68%)	150.0	181.8
Pea protein concentrate	150.0	181.8
Soy protein concentrate	150.0	181.8
Rapeseed meal	150.0	181.8
Wheat	66.0	80.0
Fish oil	10.0	12.1
DL-methionine	3.9	4.7
Yttrium oxide	0.15	0.18
Diamol	10.0	12.1
Mineral premix	10.0	12.1
**Measured nutrient content [g kg^−1^ DM]**		
Dry matter [g kg^−1^]	961	975
Crude protein (CP)	421	514
Fat	56	213
Carbohydrates	467	204
Ash	57	69
AIA	9.20	9.51
Gross energy (GE) [MJ kg^−1^ DM]	20.27	24.17
CP GE^−1^ [mg kJ^−1^]	20.76	21.25

1 Starch diet.

2 Fat diet.

### 1.4 Experimental design

The aim of the present study was to determine whether dietary composition (fat vs. starch), had an effect on chyme characteristics and the postprandial water fluxes in the gastro intestinal tract (GIT). The calculation of water fluxes was based on chyme analysis by dissection of experimental animals at three different points in time after feeding (2, 5 and 8 h). The twelve aquaria were randomly assigned to one of the two dietary treatments and within diets randomly to one of the three sampling times; resulting in a 2×3 factorial design, with two replicates per treatment (n = 2). Since the collected chyme was pooled per tank prior to analysis, the experimental unit in this study was tanks with15 fish.

### 1.5 Experimental protocol

Animals were fed restrictively during the entire experiment at approximately 80% of *ad libitum*, based on the data presented by van de Nieuwegiessen et al. [12]. Due to the difference in nutrient density of the two diets, the feeding level was based on GE instead of DM. Therefore, animals were fed isoenergetically, isonitrogeneously and received the same amount of basal ingredients, but SD fed animals had a 19.18% higher feed ration based on DM, compared to FD. The initial daily feeding ration per tank was calculated from the average animal body weight over tanks. The daily increase in feeding ratio was calculated from the expected daily animal growth, assuming a FCR of one. To ensure complete feed intake (FI) and to account for differences in feeding speed, animals were hand fed and the feeding time was recorded. To estimate FI per tank, feed refusal was weighed. Furthermore, feed spillage was collected by settling according to the procedure described by Amirkolaie et al. [11] or by netting out of the tank. The pellets were counted and their dry weight was estimated from the average pellet weight.

Chyme sampling took place on two consecutive days (34–35) and on each day, six tanks were sampled; i.e. one tank per treatment. Animals were fed a single meal and groups of 15 fish were euthanized (2- phenoxy-ethanol at 4 ml L^−1^) at 2, 5 or 8 h after feeding. Animals were weighed individually and dissected for chyme collection. Chyme was collected in four compartments of the GIT: Stomach, proximal-, mid- and distal intestine. The distinction of the gastro intestinal compartments was standardized prior to sampling, according to a subjective assessment of physiological traits (including diameter and colouration). Samples were taken quantitatively, meaning that all chyme was stripped out of each compartment. The collected samples were pooled per tank and stored in aluminium trays for the compartments stomach and mid intestine and in 50 ml plastic containers for the compartments proximal- and distal intestine. From these volumes a 0.5 ml subsample was taken for the analysis of osmolality. The wet weight of the samples was recorded before and after subsampling, to account for this loss of chyme. Prior to analysis samples were freeze-dried and ground (1.2 mm screen grinder).

### 1.6 Pellet water uptake

Water uptake of pellets during feeding has been described in several previous studies [6], [10]. A separate trial was performed in order to estimate the absorption kinetics for the two experimental diets. Therefore, 8 g of diet was placed in a spherical metal tea sieve and was submerged into 250 ml of tap water (Wageningen, The Netherlands) at room temperature (20°C) for 15, 30, 60, 120, 180 or 300s. The sieves were hung for 30 s to allow dripping and were then weighed. Samples were dried at 103°C for 3 h, cooled in a desiccator for 1 h and DW was determined. Measurements were performed in triplicate.

### 1.7 Calculations

FI [g] per tank was calculated from the daily feeding ration, subtracting the feed refusal and the feed spillage. GE intake [kJ] was calculated by multiplying the FI with the energy content of the respective diet [kJ g^−1^]. The water content of chyme [g] was calculated from the chyme wet weight and DM content and was then converted into a volumetric measure [ml], assuming that one g of water had a volume of 1 ml under the tested conditions. To calculate water fluxes it was necessary to account for the absorption of DM along the GIT. Therefore, the water content (W) [ml] of chyme samples was expressed relative to its marker content (M) [mg] as:
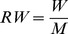
(1)


Since water fluxes in the GIT were expected to be dependent on the meal size, the relative amount of ingested DM (RDM_i_) [g DM mg^−1^ yttrium] was calculated from the ingested DM (DM_i_) on the sampling day and the marker content [mg] of the ingested feed (M) as:
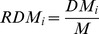
(2)


The relative water fluxes (RWF) [ml water g^−1^ ingested DM] were calculated from the relative water content (Eqn. 1) of chyme in one compartment (RW_2_), the relative water content in the previous compartment (RW_1_) and the relative ingested DM (Eqn. 2) as:
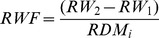
(3)


The water absorption (WA) of pellets [% of pellet weight] was calculated from the wet weight of the sample (WW_fin_) after water exposure and the initial sample wet weight (WW_ini_) as:
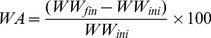
(4)


The leaching (L) of pellet DM [% of pellet weight] was calculated from the initial dry weight of the sample (DW_ini_) and the final dry weight of the sample (DW_fin_) as:
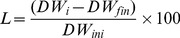
(5)


### 1.8 Analytical techniques

Due to insufficient sample material, chyme DM was assessed as freeze DW. For the measurements of osmolality chyme samples (0.5 ml) were centrifuged at 12,000 G for 10 min. The osmolality of the supernatant was determined by freezing point osmometry, in an Advanced Model 3320 Micro-Osmometer (Advanced Instruments, Norwood, MA, USA). For the analysis of yttrium, dry samples (0.3 g) were prepared by wet acid digestion. Analysis was by inductively coupled plasma atomic emission spectroscopy (ICP-OES) [13].

### 1.9 Statistics

Data was analysed using the IBM SPSS statistics software v19.0.0.1 (SPSS Inc., an IBM company, Copyright 1989–2010, Chicago, USA). Feeding time and FI data was analysed by means of One-Way ANOVA (*n* = 6, Tukey, *p*<0.05). Pellet water uptake, leaching and relative water fluxes were analysed using the linear regression function in SPSS (*p*<0.05). Slopes and intercepts of the regression analysis were compared using an ANCOVA (*p*<0.05) with the independent variable as covariate. Chyme characteristics and relative water fluxes were analysed by means of *F*-tests using a split plot model (repeated measurements ANOVA in SPSS) for the effects of diet (D), time (T), compartment of the GIT (C) and their interactions (*p*<0.05). The applied model was specified as:
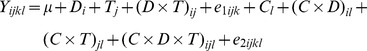
(6)Where Y_ijkl_  =  value of the chyme characteristic in diet i, at time j, replicate tank k and GIT compartment l; μ =  overall mean; D_i_  =  effect of diet i (i = 1,2); T_j_  =  effect of time j (j = 2,5,8); e_1ijk_  =  error term 1, which represents the random effect of tank k within diets and time (k = 1,2); C_l_  =  effect of compartment l (l = 1,...,4), e_2ijkl_  =  error term 2, which represents the random effect within tanks between compartments. Furthermore, main effects and interactions, were tested separately, within each compartment, by 2-way ANOVA (*n* = 2, Tukey, *p*<0.05). The assumption of normal distribution was tested with the Shapiro-Wilkinson test (*p*<0.05). All results are given as Means±SE.

## Results

Overall, both diets were readily consumed and all fish were feeding frenetically in the corner, in which the feed was supplied. Feed refusal was reported mainly in SD and at the beginning of the trial. Since animals were hand fed, feed spillage was generally low. According to subjective observations, SD groups showed a higher level of activity and aggressive behaviour. On the sampling day, FI was significantly higher (*p*<0.001) in SD (144±2 g), compared to FD (117±0 g). This is a consequence of the different energy density of the diets and the isoenergetic feeding. However, GE intake was similar for both diets (*p*>0.05) and on average 303±4 kJ. SD fed animals consumed their feeding ration significantly slower (*p*<0.001) compared to FD fed animals. Feeding times were 57.5±6.8 s and 17.7±1.3 s for SD and FD, respectively.

### 1.1 Pellet water absorption

Results for pellet water absorption of the two experimental diets are given by the linear relationships between soaking time (t) [s] and the water absorption (WA) of pellets [% of pellet weight] as:
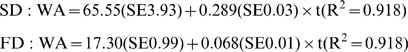



Water absorption was higher in SD compared to the FD for all measured soaking times and the intercepts were tested significantly different (*p*<0.001). The absorption of water increased with soaking time, for both diets. This increase was higher in SD compared to FD, as indicated by the significant difference in the slopes of the regression lines (*p*<0.001). Since the animals were hand fed, adapted to the feeding speed, it was assumed, that single pellets were consumed within 5 s. By extrapolation of the two linear models, previously described, the amount of water absorption during this soaking time was estimated. In SD 66.99% of the pellet weight was taken up as water, compared to 17.64% in FD. Results of the pellet water absorption trial revealed that pellet leaching was not significantly different from zero or between diets (*p*>0.05). Therefore, leaching within the considered 300 s period can be regarded as negligible and no adjustment of ingested DM or nutrient content was required.

### 1.2 Chyme osmolality

**Figure 1:**
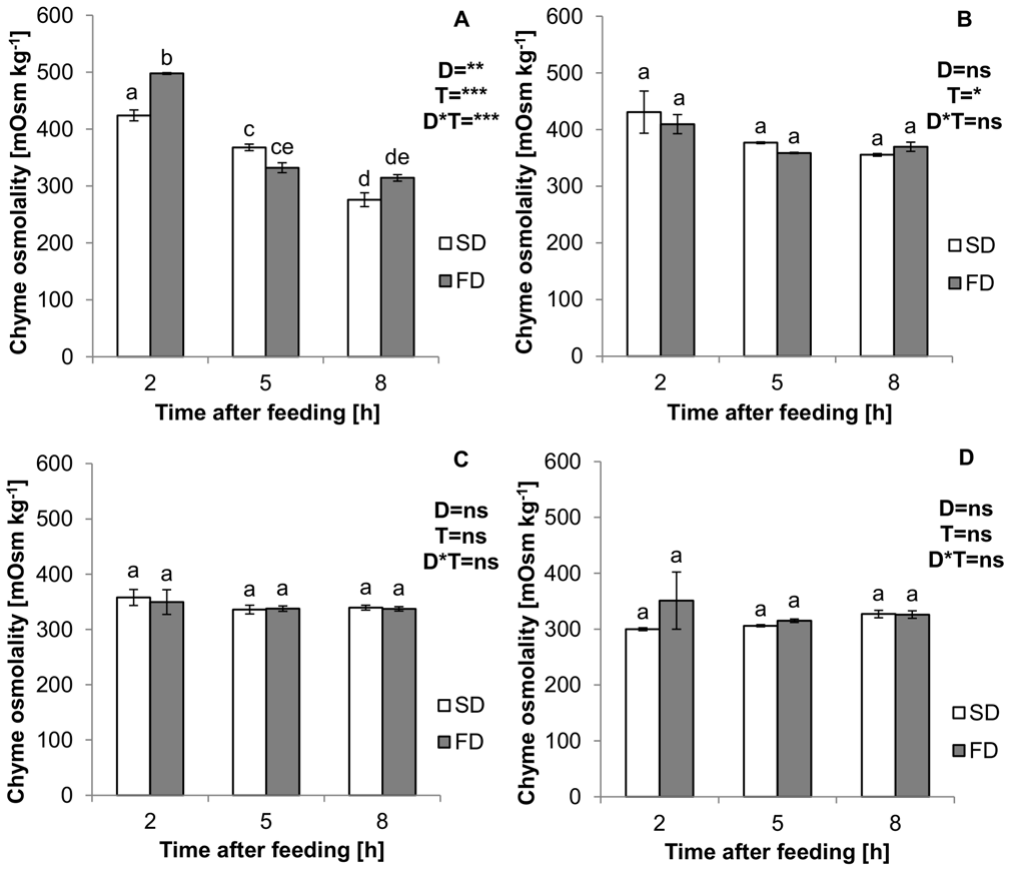
Osmolality of chyme [mOsm kg^−1^] (**Means±SE**)**.** Measured in the stomach (A), proximal intestine (B), mid intestine (C) and distal intestine (D) of African catfish (*Clarias gariepinus*) at 2, 5 and 8 h after feeding a starch (SD) or fat (FD) diet. A 2-Way ANOVA (*p*<0.05, Tukey) was used to detect the effects of diet (D), time (T) and the diet*time interaction (D*T) within compartments. Effects are marked with ns for non-significant; # *p*<0.10; * *p*<0.05; ** *p*<0.01; *** *p*<0.001. Means (*n* = 2) within compartments marked with different letters are significantly different from each other (*p*<0.05).

The results for chyme osmolality are depicted in Figure 1. Chyme osmolality was significantly different between compartments of the GIT (*p*<0.001), but not dependent on the diet (*p*>0.05). A time effect on chyme osmolality was detected (*p* = 0.002) and dependent on the compartment (*p*<0.001). The average chyme osmolality per compartment was 369±22 mOsm kg^−1^ in the stomach and increased slightly towards the proximal intestine with 383±10 mOsm kg^−1^. Thereafter, chyme osmolality decreased to 343±4 mOsm kg^−1^ in the mid intestine and 321±8 mOsm kg^−1^ in the distal intestine.

In the stomach, within compartment analysis detected significant main effects of diet (*p* = 0.008), time (*p*<0.001) and their interaction (*p* = 0.001). At 2 h after feeding chyme osmolality in the stomach was significantly higher in FD (498±2 mOsm kg^−1^), compared to SD (424±10 mOsm kg^−1^). Over time, chyme osmolality in the stomach decreased for both diets. A non-significant trend suggests that at 8 h after feeding, stomach chyme osmolality in FD was still higher compared to SD (*p*<0.10). Average chyme osmolality over diets in the stomach at 8 h after feeding was 295±12 mOsm kg^−1^. In the proximal intestine, a significant time effect was detected (*p* = 0.027), which was not influenced by the diet. Averaged over diets chyme osmolality in the proximal intestine decreased from 420±18 mOsm kg^−1^ at 2 h after feeding to 362±5 mOsm kg^−1^ at 8 h after feeding. No significant main effects or interactions were detected in the compartments mid- and distal intestine.

### 1.3 Chyme DM

**Figure 2:**
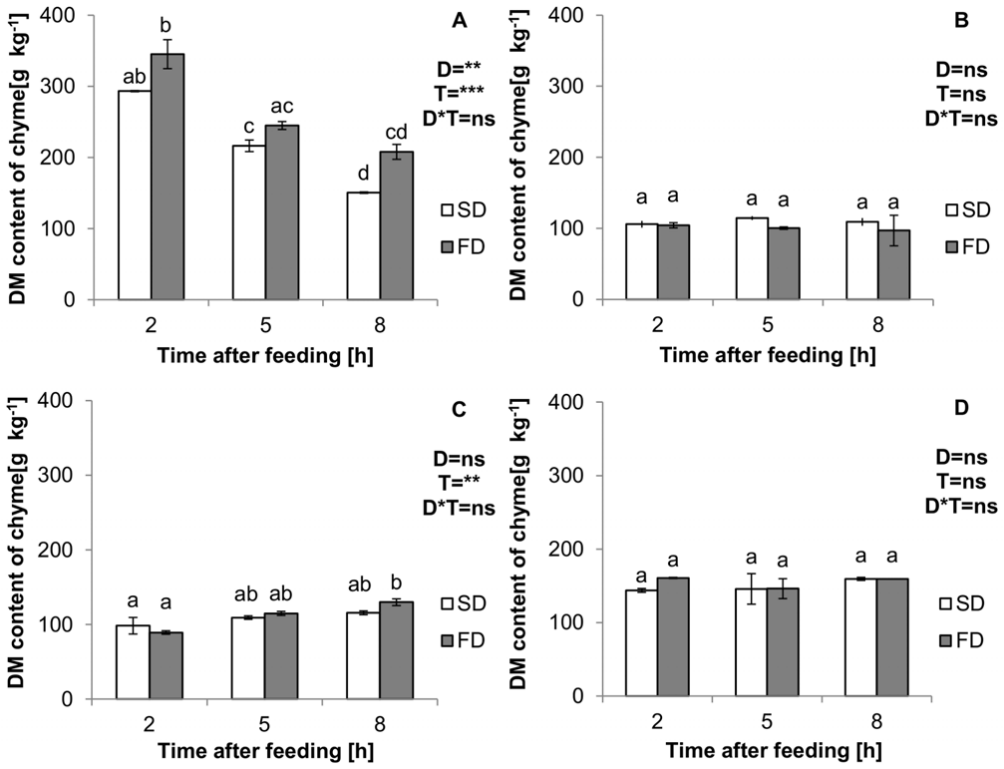
Dry matter (**DM**) **content of chyme [g kg^−1^]** (**Means±SE**)**.** Measured in the stomach (A), proximal intestine (B), mid intestine (C) and distal intestine (D) of African catfish (*Clarias gariepinus*) at 2, 5 and 8 h after feeding a starch (SD) or fat (FD) diet. A 2-Way ANOVA (*p*<0.05, Tukey) was used to detect the effects of diet (D), time (T) and the diet*time interaction (D*T) within compartments. Effects are marked with ns for non-significant; # *p*<0.10; * *p*<0.05; ** *p*<0.01; *** *p*<0.001. Means (*n* = 2) within compartments marked with different letters are significantly different from each other (*p*<0.05).

Results for the DM content of chyme are depicted in Figure 2. Chyme DM was influenced by the compartment (*p*<0.001), the diet (*p* = 0.034) and the time after feeding (*p* = 0.006). Furthermore, the compartment had an influence on the effects of diet (*p*<0.001) and time (*p*<0.001). Average DM content was highest in the stomach (243±19 g kg^−1^); decreased towards the proximal- (105±3 g kg^−1^) and mid-intestine (110±4 g kg^−1^) and increased slightly in the distal intestine (153±4 g kg^−1^).

Chyme DM in the stomach was influenced by the diet (*p* = 0.002) and averaged over time, the DM content in the stomach was 220±26 g kg^−1^ and 266±27 g kg^−1^ for SD and FD respectively. Averaged over diets chyme DM in the stomach at 2 h after feeding was 319±17 g kg^−1^ and decreased (*p* = 0.001) to 179±17 g kg^−1^ at 8 h after feeding. In all other compartments, chyme DM was similar between diets. In the mid intestine chyme DM, averaged over diets, increased (*p* = 0.004) from 93±5 g kg^−1^ at 2 h after feeding, to 123±5 g kg^−1^ at 8 h after feeding.

### 1.4 Water fluxes

**Figure 3:**
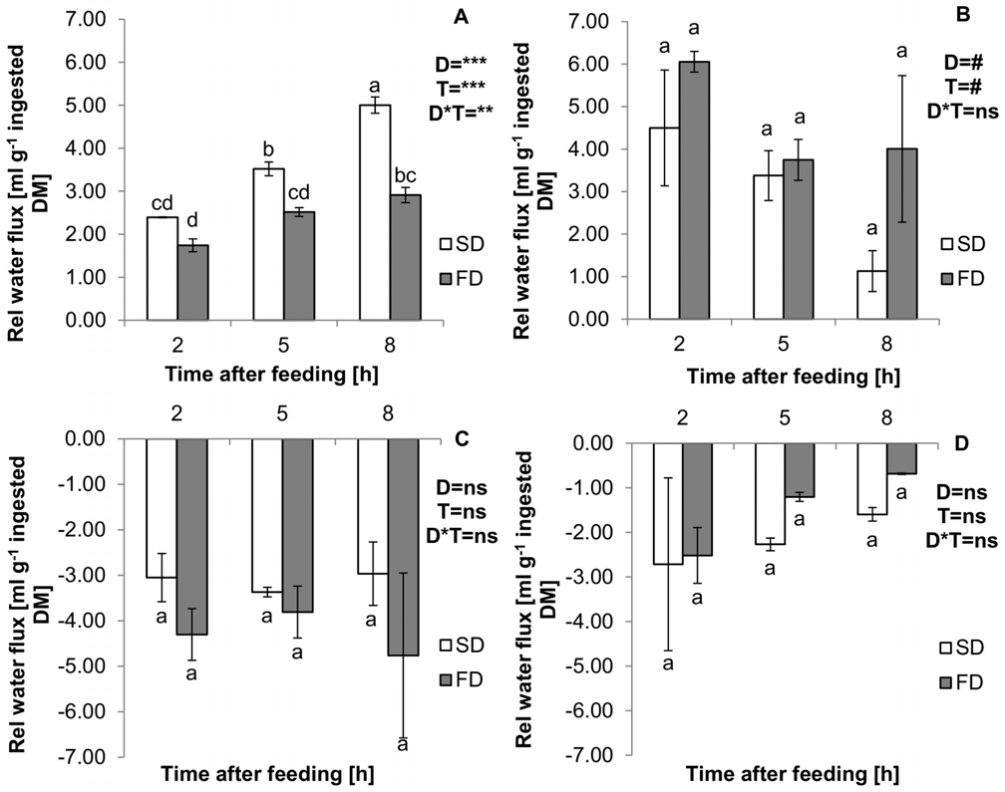
Relative water flux [ml g^−1^ ingested DM] (**Means±SE**)**.** Measured in the stomach (A), proximal intestine (B), mid intestine (C) and distal intestine (D) of African catfish (*Clarias gariepinus*) at 2, 5 and 8 h after feeding a starch (SD) or fat (FD) diet. A 2-Way ANOVA (*p*<0.05, Tukey) was used to detect the effects of diet (D), time (T) and the diet*time interaction (D*T) within compartments. Effects are marked with ns for non-significant; # *p*<0.10; * *p*<0.05; ** *p*<0.01; *** *p*<0.001. Means (*n* = 2) within compartments marked with different letters are significantly different from each other (*p*<0.05).

Results for the relative water fluxes (RWF) (Eqn. 3) in the GIT are depicted in Figure 3. Water fluxes were influenced by the compartment (*p*<0.001) and this effect was dependent on diet (*p* = 0.032) and time after feeding (*p* = 0.047). The diet did not have an influence on the calculated water fluxes (*p*>0.05). Averaged over diets and time, 3.01±0.32 ml water g^−1^ DM were added to the stomach and 3.80±0.53 ml g^−1^ DM to the proximal intestine. In the mid- and distal intestine instead, a net absorption of water took place; and relative water fluxes were −3.71±0.33 ml g^−1^ DM and −1.83±0.33 ml g^−1^ DM, respectively. The sum of relative water influx to stomach and proximal intestine was similar for both diets (One-way ANOVA, *n* = 12, *p*<0.05) and on average 6.52±0.85 ml water g^−1^ DM.

Cumulative water additions to the stomach were significantly higher (*p*<0.001) in SD fed animals (3.64±0.48 ml g^−1^ DM), compared to FD (2.39±0.23 ml g^−1^ DM). Averaged over diets, the water flux in the stomach increased (*p*<0.001) from 2.07±0.20 g g^−1^ DM at 2 h after feeding to 3.96±0.61 ml g^−1^ DM at 8 h after feeding. This time effect was dependent on the diet (*p* = 0.006), and a stronger increase in water additions to the stomach was observed in SD. When correcting the water fluxes in the stomach for the cumulative effect of this compartment, results suggest that the water addition decreased over time (*p* = 0.053). Averaged over diets the corrected water influx to the stomach decreased from 2.07±0.20 ml g^−1^ DM at 2 h after feeding, to 0.94±0.32 ml g^−1^ DM at 8 h after feeding. In the proximal intestine a non-significant trend indicates, that more water was added to FD fed animals (4.60±0.66 ml g^−1^ DM), compared to SD (3.00±0.74 ml g^−1^ DM) (*p* = 0.091). A non-significant trend suggests that water fluxes in the proximal intestine decreased over time (*p* = 0.081). Averaged over diets the water influx decreased from 5.27±0.72 ml g^−1^ DM at 2 h after feeding to 2.56±1.11 ml g^−1^ DM at 8 h after feeding. In the mid- and distal intestine, water fluxes were constant between diets and over time, since no significant main effects or interactions were detected (*p*>0.05).

**Figure 4:**
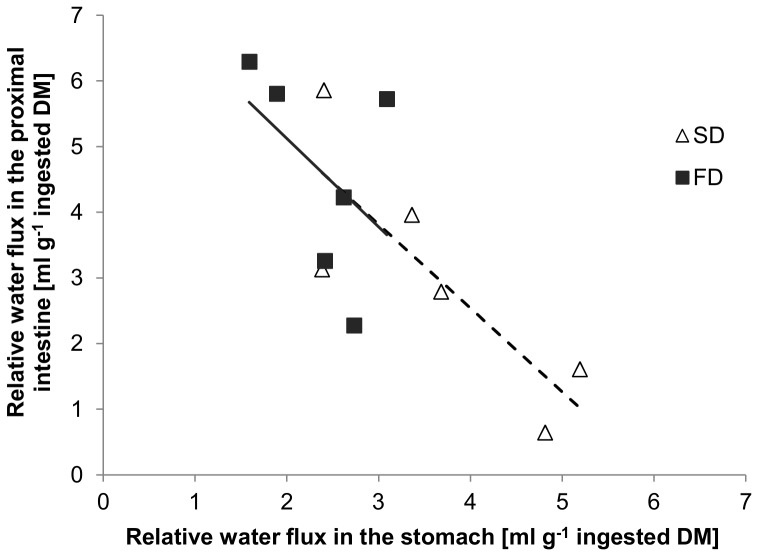
Relative water flux in proximal intestine and stomach [ml g^−1^ DM]. Relative water flux in the proximal intestine vs. Relative water flux in the stomach of African catfish (*Clarias gariepinus*) measured at 2, 5 and 8 h after feeding a starch (SD) or fat (FD) diet. The estimated linear models are given in the text. No significant differences were detected between intercepts (*p* = 0.962) or slopes (*p* = 0.959) of the linear models (One-Way ANCOVA, *n* = 2, *p*<0.05).

To better depict the relationship between the water fluxes in the stomach and proximal intestine, the relative water flux of the proximal intestine (RWF_prox_) was plotted against the relative water flux of the stomach (RWF_stom_) for both diets and a regression line was fitted (Figure 4). The linear relationship between RWF_prox_ and RWF_stom_ [ml g^−1^ DM] per diet was:



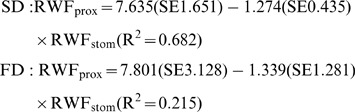



No significant differences were detected between slopes or intercepts of the two linear models (*p*>0.05). The linear relationship of the pooled data between RWF_prox_ and RWF_stom_ [ml g^−1^ DM] was:
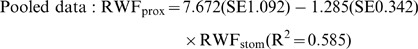



## Discussion

The aim of the present study was to detect a relationship, between dietary characteristics, i.e. the replacement of dietary fat by starch, the resulting chyme characteristics in the GIT and processes of water balance. The present results show that chyme osmolality, DM content and water fluxes were affected by the planned contrasts in dietary composition. Overall, compartments of the GIT differed significantly in chyme characteristics, which validates the division of segments made during dissection. Largest differences in chyme characteristics, between diets and over time, were observed in the stomachs of the fish. In subsequent compartments, these differences were much smaller. In general, it was observed, that the smaller compartments, proximal- and distal intestine, displayed a larger variability in the measured parameters, compared to the stomach and mid intestine. This was mainly caused by the lower amount of chyme present in these compartments, which made it increasingly difficult to sample in a quantitative way. In many fish, the distal intestine was empty. Therefore, fewer individuals contributed to the collected chyme and the effect of inter-individual differences was larger; thereby increasing variability.

The observed high initial osmotic pressure in the stomach (498±2 mOsm kg^−1^ in FD and 424±10 mOsm kg^−1^ in SD) is in line with the results obtained by Bucking and Wood [10] in their study on rainbow trout. However, they described extreme osmotic values in the stomach of up to 2.8 times plasma osmolality at 2 h after feeding. In the present study, osmolality of stomach chyme at 2 h after feeding was only 1.5 fold higher, assuming a blood plasma osmolality of approximately 300 mOsm in African catfish. A subsequent decline in chyme osmolality due to dilution by large additions of water was described in both studies. Yet, after 24 h the stomach osmolality in trout was still 1.3 fold higher than plasma values, while in the present study, stomach osmolality was close to plasma values, already at 8 h after feeding. Initial differences in stomach chyme osmolality between these two studies are likely the results of differences in dietary composition. However, the DM content of the chyme suggests that the present diets were more diluted at 2 h after feeding compared to the results presented by Bucking and Wood [10]. As described by Ruohonen et al. [7] trout experience a delay in gastric evacuation after the consumption of dry pelleted diets, which first need to be moistened in the stomach. The same is expected for African catfish, but the present results suggest that moistening of the chyme was faster compared to trout and therefore gastric evacuation was delayed to a lesser extent. However, interspecific differences in gastric evacuation rate can not be excluded and the lower rearing temperature of trout (10–13°C) [10] likely contributed to a slower gastric evacuation.

In the proximal intestine chyme osmolality increased for both diets (to 383±10 mOsm kg^−1^), and resembles the observations of Bucking and Wood [14] on rainbow trout. An increase in chyme osmolality in the proximal intestine can be explained by the secretion of bile but also pancreatic digestive fluids. Bile is known to have a higher osmolality compared to the blood plasma in trout [15] and the same is expected for African catfish. Furthermore, the enzymatic breakdown of polysaccharides in the proximal intestine yields mono- and disaccharides such as glucose and maltose. These substances are highly osmotically active and contribute to the observed increase in chyme osmolality. In fish, lipolysis in the duodenum releases mainly free fatty acids [16]. These molecules are larger than mono- or disaccharides and therefore the increase in osmolality upon hydrolysis of fat is smaller compared to starch. However, the original hypothesis that a higher dietary inclusion of starch would lead to a higher osmotic pressure in the proximal intestine was not confirmed. An explanation might be found in a higher secretion of bile in FD fed animals, due to the higher lipid content of their diet. In SD instead, it seems that free glucose (and other mono- and disaccharides) from enzymatic starch hydrolysis was readily absorbed. More herbivorous species have a higher digestive and absorptive capacity for glucose [17] and it appears that, for African catfish, this point was not exceeded by the applied starch inclusion level. The observed decrease (*p*<0.05) in chyme osmolality within the proximal intestine can be explained by a decreasing addition of bile over time [10] and a decreasing release of osmotically active molecules from the chyme.

Dissection of the experimental animals at different time points after feeding allowed calculating the relative water fluxes (Eqn. 3) along the GIT in response to feeding. Present results show that large amounts of water were added to the stomach and proximal intestine, while in the mid- and distal intestine reabsorption of water took place. Similar results were reported by Bucking and Wood [10], who calculated the postprandial water fluxes in the GIT of rainbow trout; but in addition, the present setup allowed assessing the response of these water fluxes to dietary composition. Results indicate, that during the first 8 h after feeding, significantly more water was added to the stomach of animals fed a higher dietary starch content (Figure 3 A). Interestingly, a trend (*p*<0.10) suggests that in the proximal intestine, the opposite was the case, and the reported water influx was numerically higher in FD (Figure 3 B). By plotting the relative water flux of the proximal intestine versus the relative water flux of the stomach a significant negative relationship was detected (Figure 4). The present data suggests that 59% of the water influx to the proximal intestine can be explained by the previous addition to the stomach. In other words, a surplus water addition to the stomach was compensated by a decreased addition to the proximal intestine. And in fact, the sum of water additions to both compartments, was not influenced by the consumed diet (*p*>0.05) and on average 6.52±0.85 ml water were added to every g ingested DM.

In the present study, it was not possible to differentiate between endogeneous and exogeneous water additions to the stomach, since no marker was added to the system water (as performed by Kristiansen and Rankin [6]). Nevertheless, the addition of water to the stomach until 2 h after feeding was not significantly different between diets. Furthermore, the cumulative water influx to the stomach increased faster for SD, compared to FD (*p*<0.01). This illustrates that the observed differences in water fluxes in the stomach, between diets, can be attributed to postprandial water additions. According to Bucking and Wood [10], the osmotic pressure in the stomach, is the main driver for endogeneous water additions to this compartment. Also in the present study, indications were found that substantiate this assumption and in fact, most water was added to the stomachs at the time of highest chyme osmolality. Interestingly, the surplus addition of water to the stomachs in SD was not accompanied by a higher osmolality of chyme. However, it seems possible that an increase in the concentration of osmotically active substances in the chyme, due to hydrolysis of macromolecules, drew an equivalent amount of water into the stomach and thereby prevented an increase in chyme osmolality. This is substantiated by the lower (*p*<0.01) DM content of the stomach chyme in SD.

Considering that the outcome of the digestive processes in the stomach was a chyme of similar characteristics, it can be speculated that fish were able to actively control the addition of fluid to the stomach. Due to the large osmotic gradient available to drive an endogeneous water influx, it is questionable whether active transport mechanisms are a sound pathway. Therefore, it is suggested that in the same way as marine species drive water uptake in the intestine, both marine and FW species could regulate the influx of interstitial water to the stomach. By actively controlling the osmolality of the lateral interspace, the osmotic gradient across the epithelium could be adapted to the required osmotic water influx to the stomach; lateral Na^+^/K^+^-ATPase, Na^+^/H^+^-exchangers and Cl^−^-channels are suggested as regulating units. The involvement of some type of mitochondria rich cell seems likely and could be substantiated in immunocytochemical studies. According to recent findings, the water flux is likely via the cellular pathway, involving aquaporins [18].

Postprandial drinking is another way, in which the animals can regulate the water addition to the chyme [4], [5], [6], [7]. However, it remains unclear, what triggers these drinking events. In euryhaline fish, drinking represents the most immediate reaction to changes in osmotic conditions and is strongly regulated by anti-dipsogenic (thirst supressing) hormones [19]. In fish, reflex swallowing is triggered by the hindbrain, often in response to hypovolemia (state of decreased blood volume) [20]. Bucking and Wood [10] described an increase in the plasma osmolality of trout, after feeding. This was attributed to a combination of osmotic water additions to the chyme and the absorption of nutrients in the intestine. A higher plasma osmolality could be an indicator for an on-setting hypovolemia, which concentrates the blood plasma and could therefore trigger drinking. This mechanism would function as a feedback loop between endogeneous and exogeneous water additions to the stomach, likely via the action of angiostenin II, a strong dipsogen, which acts on the area postrema, as part of the renin-angiostenin system [21], [22]. The result is a combination of endogeneous and exogeneous, postprandial water additions to the stomach, which Bucking and Wood [10] considered a likely explanation for their results. In a study on juvenile trout, Kristiansen and Rankin [6] incorporated a non-absorbable marker (^51^Cr-EDTA) into the ambient water during feeding and were able to discriminate between different water flows. They identified 25–35% of stomach water influx, to be exogeneous, while 34–44% was endogeneous. Despite the large inter individual differences described, convincing evidence remains that fish are able to combine different water sources, in order to establish the required water content of chyme, for stomach evacuation and subsequent digestion.

Unlike in the stomach, the measurement of water fluxes in the proximal intestine is not immediately subjected to a bias from exogeneous water fluxes. Water additions to the proximal intestine are expected, mainly from those digestive fluids secreted by the pancreas and the gall bladder [10]. The higher water flux in FD is likely due to an increased bile secretion in response to the higher dietary lipid level [16]. The decrease (*p*<0.10) in water additions over time can be explained by a decreasing secretion of bile and an increased absorption of water in the proximal intestine [23] [10]. Another source of water additions to the proximal intestine is the evacuation of liquid from the stomach [10]. Since the used yttrium oxide is an insoluble marker, a basic assumption for the estimation of water fluxes was that water and chyme display similar passage rates. However, it is possible that the chyme of the two dietary treatments differed in water uptake behaviour, as a results of the developed chyme characteristics. The higher lipid content in FD might have restricted the mixing of water and chyme in the stomach. The formation of a distinct liquid phase and its evacuation would lead to an underestimation of the water addition to the stomach and an overestimation of the water addition to the proximal intestine. In FD fed animals, the chyme absorbed most water in the proximal intestine, which is striking for such a short compartment, in which the chyme will only reside for a limited time. This was likely due to the emulsifying action of the added bile, which mediated a water uptake into the hydrophobic chyme. In contrast, in SD, a higher viscosity and water retention capacity of extruded maize [24] made a separation of the stomach chyme into a solid and a liquid phase less likely. Consequently, a more accurate estimation of the real water fluxes is expected in SD. Bucking and Wood [10] combined ballotini beads with a water-soluble marker (^3^[H]-PEG 4000) to detect different passage rates of the solid and liquid phase of the chyme. Their results indicate that at determined points in time, the soluble marker was evacuated from the stomach at a higher rate than the beads and similar results have been described in studies on mammals [25], [26], [27]. For their results on trout, Bucking and Wood [10] concluded that the slightly faster evacuation of the soluble marker from the stomach had only a minor impact on the outcome of the calculated water fluxes. Due to the different dietary composition and species, it is difficult to make comparisons to the present study. Further research on the discrimination between endogeneous and exogeneous water additions to the chyme and the dissociation of chyme into fractions with different passage rates, in relation to dietary composition and resulting chyme characteristics, is required.

In the mid- and distal intestine, water absorption took place at a similar rate for both diets (Figure 3 C and D). When balancing the water fluxes over the entire GIT, it was observed that for both diets, more water was added to the chyme compared to reabsorbed amounts. This led to the rectal excretion of 1.32±0.34 ml water g^−1^ DM in SD and 1.23±0.24 ml g^−1^ DM in FD. Considering the implications of a hyperosmoregulatory strategy, rectal water excretion can generally be considered advantageous. But a net excretion will only take place, if water additions to the GIT were largely from endogeneous sources. Bucking and Wood [10] found indications for a net excretion of water with the faeces in rainbow trout. In light of the results obtained by Kristiansen and Rankin [6] on trout, it seems likely that water additions are at least partly of exogeneous origin. When assuming that only 45% of the stomach influx was endogeneous, the net water balance for the present study would be close to zero. Yet, this does not mean that no osmotic challenge is imposed on the fish, when consuming dry pelleted feeds, since the times of highest water secretion and absorption can differ significantly [10].

## Conclusion

In the present study, the planned contrasts in dietary composition, i.e. the replacement of dietary fat by starch had an influence on chyme characteristics and processes of water balance in the GIT. Largest differences in chyme characteristics between diets were observed in the stomach and decreased towards the distal GIT. A high osmotic pressure in the stomach was detected for both diets and is suggested to drive the addition of endogenous water to the chyme. Large additions of water were observed in the stomach and proximal intestine for both diets, while absorption of water took place in the mid and distal intestine. Water balance in the GIT was altered by the consumption of the two experimental diets and present results suggest that the isoenergetic replacement of fat by starch induced a shift in water secretion from the proximal intestine to the stomach. Nevertheless, the overall addition of water to the GIT was not affected by the dietary treatment. For future studies on water fluxes in the GIT of fish, it is recommended to perform feeding trials with a combination of inert markers, as applied by other authors. This will allow accounting for the origin of the water added to the stomach, as well as for differences in passage rate of the liquid and solid phase of the chyme.

## References

[1] Marshall WS, Grosell M (2006) Ion transport, osmoregulation and acid–base balance. In: Claiborne DEaJB, editor. The Physiology of Fishes. 3 ed. BocaRaton: CRC Press. pp. 177–230.

[2] Heisler N (1984) Acid-base regulation in fish. In: Hoar WS, Randall DJ, editors. Fish Physiology. New York: Academic Press. pp. 315–401.

[3] Wood CM, Bucking C (2010) The role of feeding in salt and water balance. In: Grosell M, Farrell AP, Brauner CJ, editors. Fish Physiology The multifunctional gut of fish. London, UK: Academic Press. pp. 165–212.

[4] Smith HS (1930) The absorption and excretion of water and salts by marine teleosts. Am J Physiol 93: 480–505.

[5] Fuentes J, Eddy FB (1997) Drinking in marine, euryhaline and freshwater teleost fish. In: Hazon N, Eddy FB, Flik G, editors. Ionic Regulation in Animals. New York: Spring-Verlag. pp. 135–149.

[6] Kristiansen HR, Rankin JC (2001) Discrimination between endogeneous and exogeneus water sources in juvenile rainbow trout fed extruded dry feed. Aquatic Living Ressources 14: 359–366.

[7] Ruohonen K, Grove DJ, McIlroy JT (1997) The amount of food ingested in a single meal by rainbow trout offered chopped herring, dry and wet diets. Journal of Fish Biology 51: 93–105. 9236091 10.1111/j.1095-8649.1997.tb02516.x

[8] Buddington RK, Krogdahl A, Bakke-McKellep AM (1997) The intestines of carnivorous fish: Structure and functions and the relations with diet. Acta Physiologica Scandinavica 161. 9421581

[9] Windell JT, Norris DO, Kitchell JF, Norris JS (1969) Digestive response of rainbow trout, *Salmo gairdneri*, to pellet diets. Journal of the Fisheries Research Board of Canada 26: 1801–1812.

[10] Bucking C, Wood CM (2006) Water dynamics in the digestive tract of the freshwater rainbow trout during the processing of a single meal. Journal of Experimental Biology 209: 1883–1893. 16651554 10.1242/jeb.02205

[11] Amirkolaie AK, Verreth JAJ, Schrama JW (2006) Effect of gelatinization degree and inclusion level of dietary starch on the characteristics of digesta and faeces in Nile tilapia (Oreochromis niloticus (L.)). Aquaculture 260: 194–205.

[12] van de Nieuwegiessen PG, Olwo J, Khong S, Verreth JAJ, Schrama JW (2009) Effects of age and stocking density on the welfare of African catfish, Clarias gariepinus Burchell. Aquaculture 288: 69–75.

[13] van Bussel W, Kerkhof F, van Kessel T, Lamers H, Nous D, et al (2010) Accurate Determination of Titanium as Titanium Dioxide for Limited Sample Size Digestibility Studies of Feed and Food Matrices by Inductively Coupled Plasma Optical Emission Spectrometry With Real-Time Simultaneous Internal Standardization. Atomic Spectroscopy 31: 81–88.

[14] Bucking C, Wood CM (2006) Gastrointestinal processing of monovalent ions (Na^+^,Cl^−^, K^+^) during digestion: implications for homeostatic balance in freshwater rainbow trout. Am J Physiol 291: R1764–R1772. 10.1152/ajpregu.00224.2006 16902189

[15] Grosell M, O'Donnell MJ, Wood CM (2000) Hepatic versus gallbladder bile composition – *in vivo* transport physiology of the gallbladder in the rainbow trout. Am J Physiol 278: R1674–R1684. 10.1152/ajpregu.2000.278.6.R1674 10848538

[16] Bakke AM, Glover C, Krogdahl A (2010) Feeding Digestion and Absorption of Nutrients. In: Grosell M, Farrell AP, Brauner CJ, editors. Fish Physiology The multifunctional gut of fish. London, UK: Academic Press. pp. 56–108.

[17] Stone DAJ (2003) Dietary carbohydrate utilization by fish. Rev Fish Sci 11: 337–369.

[18] Wood CM, Grosell M (2012) Independence of net water flux from paracellular permeability in the intestine of *Fundulus heteroclitus*, a euryhaline teleost. Journal of Experimental Biology 215: 508–517. 22246259 10.1242/jeb.060004

[19] Takei Y (2002) Hormonal control of drinking in eels: an evolutionary approach. In: Hazon N, Flik G, editors. Osmoregulation and Drinking in Vertebrates. Oxford: BIOS Scientific Publishers Ltd. pp. 61–82. 14992145

[20] Takei Y, Balment RJ (2008) The neuroendocrine regulation of fluid intake and fluid balance. In: J. Bernier N, Van Der Kraak G, Farrell AP, Brauner CJ, editors. Fish Neuroendocrinology. San Diego: Academic Press. 365–419.

[21] Takei Y, Hirano T, Kobayashi H (1979) Angiotensin and water intake in the Japanese eel, *Anguilla japonica* . Gen Comp Endocrinol 38: 446–475. 10.1016/0016-6480(79)90155-2 478280

[22] Nobata S, Takei Y (2011) The area postrema in hindbrain is a central player for regulation of drinking behavior in Japanese eels. American Journal of Physiology – Regulatory Integrative and Comparative Physiology 300: 1569–1577. 10.1152/ajpregu.00056.2011 21451142

[23] Bogé G, Lopez L, Peres G (1988) An in vivo study of the role of the pyloric caeca in water absorption in rainbow trout (*Salmo gairdneri*). Comp Biochem Physiol 91: 9–13.

[24] Anguita M, Gasa J, Martín-Orúe SM, Pérez JF (2006) Study of the effect of technological processes on starch hydrolysis, non-starch polysaccharides solubilization and physicochemical properties of different ingredients using a two-step in vitro system. Animal Feed Science and Technology 106: 99–115.

[25] Faichney GJ, Beever DE, Black JL (1980) Prediction of the fractional rate of outflow of water from the rumen of the sheep. Agric Syst 6: 261–264.

[26] Faichney GJ, White GA (1988) Rates of passage of solutes, microbes and particulate matter through the gastrointestinal tract of ewes fed at a constant rate throughout gestation. Aust J Agric Res 39: 481–488.

[27] Owens FN, Goetsch AL (1988) Ruminal fermentation. In The Ruminant Animal: Digestive Physiology and Nutrition; Church DC, editor. Englewood Cliffs NJ: Prentice-Hall. 145–167 p.

